# Ultrasound microbubbles mediated miR-let-7b delivery into CD133^+^ ovarian cancer stem cells

**DOI:** 10.1042/BSR20180922

**Published:** 2018-09-28

**Authors:** Chaopin Yang, Bingcheng Li, Jinsui Yu, Feng Yang, Kuan Cai, Zhiyi Chen

**Affiliations:** Department of Ultrasound Medicine, Laboratory of Ultrasound Molecular Imaging, The Third Affiliated Hospital of Guangzhou Medical University, Guangzhou 510150, People’s Republic of China

**Keywords:** CD133+, miR-let-7b, Ovarian cancer stem cell, SonoVue, Therapy, Ultrasound

## Abstract

Ovarian cancer stem cells (OCSCs) are considered the reason for ovarian cancer’s emergence and recurrence. Ultrasound-targetted microbubble destruction (UTMD), a non-vial, safe, and promising delivery method for miRNA, is reported to transfect cancer stem cells (CSCs). In the present study, we investigated to transfect miR-let-7b into OCSCs using UTMD. The CD133^+^ OCSCs, accounted for only 0.1% of ovarian cancer cell line A2780, were separated by flow cytometry, and the CSC characteristics of CD133^+^ OCSCs have been proved by spheroid formation and self-renewal assay. The miR-let-7b transfection efficiency using UTMD was significantly higher than other groups except lipofectamine group through flow cytometry. The cell viability of all groups decreased after transfection, and the late apoptosis rate of CD133^+^ OCSCs after miR-let7b transfection induced by UTMD was 2.62%, while that of non-treated cells was 0.02% (*P*<0.05). Furthermore, the Western blot results demonstrated that the stem cells surface marker of CD133 expression has decreased. Therefore, our results indicated that UTMD-mediated miRNA delivery could be a promising platform for CSC therapy.

## Introduction

Ovarian cancer is one of the three malignant tumors of female reproductive system, of which 70% is epithelial ovarian cancer, which is characterized with an initial asymptomatic period and aggressive growth [1]. However, few effective screening tests exist, and thus, approximately 70% of women with advanced ovarian cancer (stage III or IV) have a poor prognosis with present therapies (mainly consisting of surgery, chemotherapy, and radiotherapy), and its 5-year survival rate is less than 30% [2]. The poor treatment outcome for ovarian cancer is due to ovarian cancer stem cells (OCSCs) with high recurrence and chemotherapy resistance. Therefore, it is significant to explore innovative and effective treatment methods aiming at OCSCs.

In recent years, the concept of the cancer stem cells (CSCs) has been introduced with in-depth study on tumorigenesis and developmental mechanism. In 2001, Reya et al. [3] put forward the CSC theory and proved the presence of CSCs in leukemia. They demonstrated that CSCs are a small group of cells capable of self-renewal and multidirectional differentiation in the tumor tissue and have stronger tumorigenic ability than the other tumor cells. At present, CSCs have been successfully separated from breast cancer [4], glioma [5], lung cancer [6], and colorectal cancer [7]; the genesis, metastasis, and drug resistance of CSC has also been confirmed. Thus, CSC is a promising therapy target in tumor therapy research.

Effective separation of CSCs is the premise for OCSC-targetted therapy research. The most widely used methods for CSC separation include flow cytometry sorting and magnetic bead selection with specific, high expression cell surface markers, such as CD133, CD44, CD117, and CD24 [8]. Amongst these surface markers, CD133 (prominin-1) is one of the most dependable surface markers for CSC. Baba et al. [9] first tested CD133^+^ ovarian cancer cells properties, such as drug resistance, *in vitro* differentiation ability, and *in vivo* tumorigenicity. The results showed that the CD133^+^ cell population has CSC characteristics of strong proliferation and multidirectional differentiation ability. Cioffi et al. [10] also demonstrated that the CD133^+^ ovarian cancer cells have stronger drug resistance, tumor metastasis, and CSC sphere formation than CD133^−^ ovarian cancer cells. Moreover, CD133^+^ ovarian cancer cells have shown much stronger transplanted capacity (cells forming transplanted tumor) than CD133^−^ cells. Together, these findings suggest that CD133^+^ ovarian cancer cells are OCSCs and can be used in OCSC therapy experiments.

miRNAs [11] play an important role in tumorigenesis, metastasis, drug resistance, and recurrence of CD133^+^ OCSCs [12]. The highly conserved miRNA, miR-let-7b, has been reported as a tumor suppressor gene that forms a dual negative feedback loop with Lin28. miR-let-7b always has low expression in tumors, and an increase in expression level can inhibit the proliferation and growth of CD133^+^ OCSCs, thus enhancing the effect of radiotherapy and chemotherapy [13,14]. Therefore, our study intends to synthesize miR-let-7b expression vectors and promote their expression in CD133^+^ OCSCs to achieve the *in vitro* treatment of ovarian cancer [15].

To date, gene delivery methods include physical, chemical, and viral approaches. Viral transfection has a high transfection efficiency, but suffered from the tendency to be randomly integrated into the host, stable long-term expression, immunogenicity, and potential carcinogenicity, which limits its clinical applications. Micro-injection, electroporation, and particle bombardment are also limited to their complex operation or continued exogenous gene expression. Chemical transfection methods using cationic polymers or liposomes have defects of great cytotoxicity, poor targetting, and unstable gene expression despite low immunogenicity [16,17]. Therefore, a safe and efficient gene targetting delivery method is needed.

Ultrasound-targetted microbubble destruction (UTMD) has been proven as a promising gene delivery method in many studies [18]. The cavitation effect induced by UTMD is divided into inertial cavitation and transient cavitation, which have different biological effects on the cells. Under steady-state cavitation, the mechanical vibration and volume changes of contrast agent can not only change the cell membrane potential through ion concentrations but also produce shear force on the cell membrane and stimulate cell endocytosis, thus promoting cross-cell membrane gene transport [19]. Transient cavitation induced by UTMD can also cause cell membrane potential changes, instantaneous shear force, a strong shock and thermal effect, which can destroy the cell integrity and transport macromolecules through intercellular space [20]. Thus, UTMD mainly relies on these two mechanisms to facilitate the cells to take in genes [21] by endocytosis and sonoporation.

In this work, ovarian cancer cells were isolated from the human ovarian cancer cell line A2780 with OCSCs surface marker CD133, and identified as stem-like cells in OCSCs. Then the effects of targetted gene delivery of miR-let-7b through UTMD to CD133^+^ OCSCs were explored.

## Materials and methods

### Isolation and culture of CD133^+^ OCSCs

Ovarian cancer A2780 cells (Shanghai Zhichenhui Biology Co., Ltd.) were cultured in the complete DMEM/F12 culture medium (Gibco) and dissociated using trypsin-EDTA (prepared into cell suspension and centrifuged to form a cell pellet after the supernatant was discarded). The cell pellet was washed with 2 ml PBS twice, and 1 μl APC marked murine anti-human CD133 antibody (APC-CD133) (eBioscience) was added into the cell suspension and incubated at 4°C for 30 min. The cells were washed twice with PBS and analyzed by flow cytometry (BD FACS Aria) to get CD133^+^ ovarian cancer cells.

### Characterization of CD133^+^ OCSCs

#### Spheroid formation ability

The CD133^+^ OCSC cell pellet was collected in the complete culture medium and re-suspended with serum-free medium containing EGF and bFGF (PeproTech). The suspension was placed at 10^4^ cells per well on to a low-adhesion six-well plate. The morphological characteristics of CD133^+^ OCSCs were observed in the serum-free medium.

#### Self-renewal ability

The serum-free cultured CD133^+^ OCSCs were collected by centrifugation at 800 rpm for 4 min. The supernatant was removed and then the serum-free medium was added to resuspend the cells. The culture continued until spheroid cells were formed.

#### Differentiation ability

CD133 expression was determined by FACS. CD133^+^ OCSCs were cultured in complete culture medium for 2 weeks, centrifuged, and tested for the differentiation ability of CD133^+^ OCSCs by qPCR experiment.

### Transfection groups and methods

The experiment groups were as follows: (i) the control group (C), (ii) the plasmid group (P), (iii) the ultrasound irradiation + plasmid group (US+P), (iv) the SonoVue+plasmid group (MB+P), (v) the ultrasound irradiation + SonoVue + plasmid group (UTMD+P), and (vi) the liposome + plasmid group (L+P). Ultrasound irradiation parameters (Metron) were 1 MHz transducer frequency, 10% duty cycle, 1 W/cm^2^ irradiation intensity, and 1 min irradiation time. CD133^+^ OCSCs in logarithmic growth phase were collected and transferred into a 24-well culture plate with cell density of 10^5^/well with 400-μl DMEM (excluding FBS and double antibody) per well. Before ultrasound irradiation, SonoVue microbubble (Braco) suspension (80 μl/well) and plasmid solution (15 μg/well) were mixed and incubated. The microbubble/plasmid mixture, the plasmid solution, or SonoVue was added into each well according to the experiment groups. The ultrasonic probe was fixed at the bottom of the irradiation sink, and the irradiation hole was aligned with the center of the probe. For L+P group, the transfection was performed by using Lipofectamine 2000 (Invitrogen, SanDiego, CA, U.S.A.) according to the manufacturer’s instructions. The miR-let-7b plasmid and Lipofectamine 2000 were diluted into 50 μl Opti-MEM medium respectively for 5 min. Then the diluted miR-let-7b and Lipofectamine 2000 were mixed for 20 min. And the mixture was added into plates as the other groups. After 4 h of culture, the complete culture medium of all groups (including 10% FBS but excluding penicillium and streptomycin) was used for the replacement of Opti-MEM for the next culture.

### Expression of EGFP protein in CD133^+^ OCSCs observed by fluorescence microscope

The expression of EGFP protein in CD133^+^ OCSCs was triggered by blue exciting light after 48 h transfection and observed under a fluorescence microscope (Olympus). First, the focal length was accurately adjusted under the fluorescence condition and the cell growth was observed roughly. Then, the expression of EGFP protein in CD133^+^ OCSCs was observed under fluorescence, and bright field photographs were acquired.

### Test of CD133^+^ OCSC gene transfection efficiency

The CD133^+^ OCSCs were collected after 48 h transfection. The cells were dissociated by trypsin/EDTA for 2 min and the complete culture medium was added to stop trypsinization. The cells were centrifuged and re-suspended in PBS solution. They were screened by a filter with an aperture of 70 μm to yield the flow samples. The samples were placed in the sampling room of flow cytometer to test EGFP expression with a 488-nm laser. The non-transfected cells in the control group were used as negative controls.

### Cell viability after CD133^+^ OCSC transfection

The CD133^+^ OCSCs after 48 h transfection were inoculated into a 96-well plate with the same method as stated above. The 96-well plate was then incubated in the dark for 1–2 h, and the absorbance at 450 nm of each group was measured by a microplate reader. The cell survival rate of each group = (OD of the experiment group − OD of the control group)/(OD of the control group − OD of the blank control group).

### miR-let-7b expression in CD133^+^ OCSC after transfection

To assess miR-let-7b expression in CD133^+^ OCSCs, qRT-PCR analysis was performed with internal standards. Total RNA was isolated from the harvested cells and reverse transcribed into cDNA with the RevertAid Reverse Transcriptase (Thermo Fisher Scientific) according to the manufacturer’s instructions. qRT-PCR was performed using the MicroRNAs Quantitation PCR Kit (Sangon Biotech). The relative expression of miR-let-7b relative to non-treated cells was calculated by the ΔΔ*C*_t_ method in triplicate experiment.

### Apoptosis of CD133^+^ OCSC assay after transfection

Propidium iodide (PI) (BD Pharmingen, U.S.A.) was used to evaluate the late apoptosis of CD133^+^ OCSC after a 48 h miR-let-7b transfection. Briefly, the treated cells were harvested, double-washed with PBS, and then fixed by cold ethyl alcohol for 2 h. Then, the treated cells were suspended in PBS for 5 min, and 1 ml PI was added to the cell suspension for 15 min at 4°C in the dark. Finally, samples were analyzed by a BD Accuri C6 flow cytometer (BD Biosciences, U.S.A.).

### Protein expression of specific genes of tumor stem cells

The OCSC protein expression mediated by miR-let-7b was detected by Western blot analysis. Cells after 48 h transfection were harvested and lysed in cold RIPA buffer (Thermo Fisher Scientific, U.S.A.) with a protease inhibitor (Omplete Mini, Roche). Then, the lysates were incubated at 4°C for 15 min and centrifuged at 13000 rpm for 15 min. The protein concentration of the supernatants was determined with the Bicinchoninic Acid Protein Assay Kit. After heat denaturation, the protein samples were loaded into SDS/PAGE and transferred on to nitrocellulose membrane (Amersham Hybond ECL, GE Healthcare). The blotted membranes were immunostained with antibodies directed against CD133/1 (W6B3C1, Miltenyi Biotec, U.S.A.). This signal was visualized by Beyo chemiluminescence (ECL) according to the manufacturer’s protocol and quantitated using the ImageJ software.

### Statistical analysis

Statistical software SPSS 16.0 (SPSS Inc., Chicago, U.S.A.) was used for data analysis and the measurement data were expressed as the mean ± S.D. (x ± SD). A *t*test was performed for two groups of data, while one-way ANOVA was performed for multiple groups of data. *P*<0.05 indicated statistically significant differences.

## Results

### Sorting of CD133^+^ OCSC

**Figure 1:**
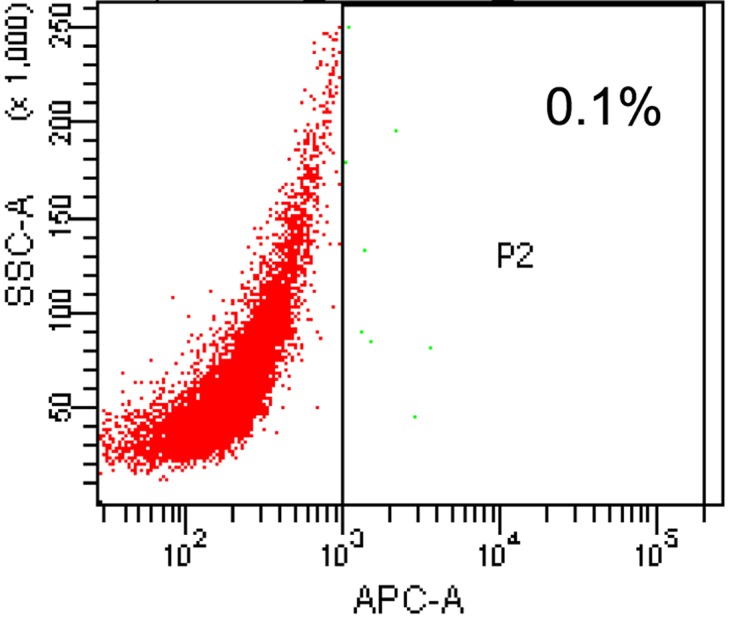
Sorting of CD133^+^ OCSC by a flow sorter

The ovarian cancer A2780 cells were marked by APC-CD133 and the CD133^+^ OCSC were sorted by a flow sorter. The sorting result showed that CD133^+^ OCSCs accounted for only 0.1% of A2780 cells (Figure 1).

### Characteristic tests of CD133^+^ OCSCs

#### Spheroid formation assay

**Figure 2:**
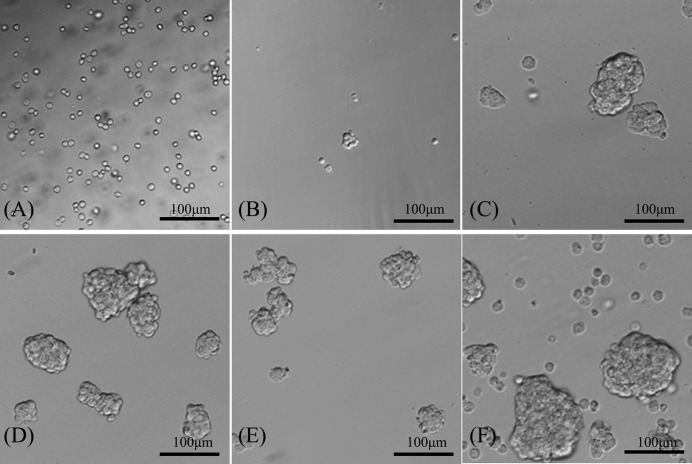
CD133^+^ OCSC spherical cells (100×) (**A**–**F**) represent the first to sixth day of serum-free culture.

The CD133^+^ OCSCs suspended in the serum-free culture medium and were observed under an optical microscope. After 48 h of suspension culture, some of the suspended cells were observed to cluster and grow spherically. However, the spheroids were small in number and volume, and were composed of three to five cells with consistent sizes. They were 3D and had light refractivity. After culturing in the serum-free medium for 5–6 days, the number and volume of spheroids were considerably greater and each one was composed of dozens of cells, which appeared round or polygonal in shape (Figure 2).

The spherical cells cultured in serum-free medium were observed under an optical microscope, showing closer intercellular junctions and indefinite boundaries. The spherical cells had high central cell density, low peripheral cell density, poor light transmission of central cells, and strong translucency of peripheral cells.

#### Self-renewal ability test

**Figure 3:**
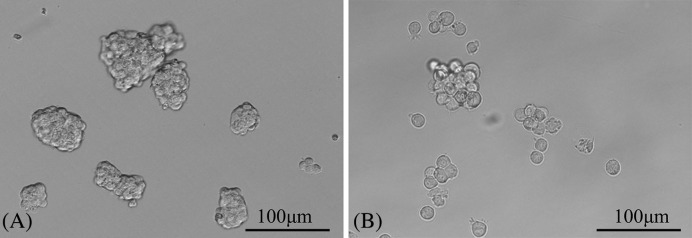
Microscopic observations of CD133^+^ OCSCs before and after passage (100×) (**A**,**B**) were the microscopic observations of CD133^+^ OCSCs growing into spherical cells in the serum-free culture medium before and after passage. It could be observed that the spherical cells were further cultured into single cells after mechanical blowing, and the single cells returned to be spheroids after 2 days.

After culturing in the serum-free culture medium, CD133^+^ OCSCs formed spheroids stably. The spheroids then were processed into single cells through mechanical blowing or digestion in tryplE solution and were cultured with the serum-free medium into two different flasks or plates. After a 2-day passage, the spheroidization of single cells was observed, and it could be found that the single cells still cultured into spheroids (Figure 3).

#### Differentiation characteristics

**Figure 4:**
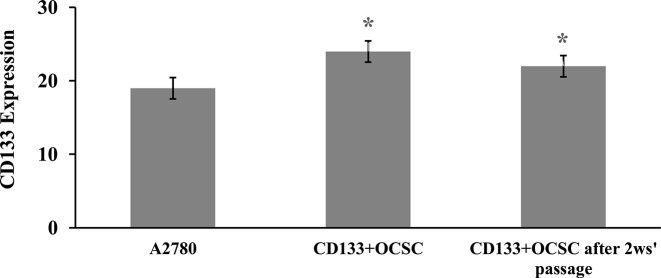
Expression of CD133 before and after the passage of CD133^+^ OCSCs *: Difference between the surface expression of CD133 between fresh CD133^+^ OCSCs and CD133^+^ OCSCs after 2-week passage (*P*<0.05).

The CD133 expression in A2780 cells, CD133^+^ OCSCs and CD133^+^ OCSCs cultured for 2-week passage was tested by qPCR. The results showed that CD133^+^ OCSCs had higher CD133 expression than A2780 cells and CD133^+^ OCSCs for the 2-week passage. Furthermore, the CD133 expression of CD133^+^ OCSCs after 2-week passage was significantly lower than CD133^+^ OCSCs (Figure 4).

### EGFP observations after transfection

**Figure 5:**
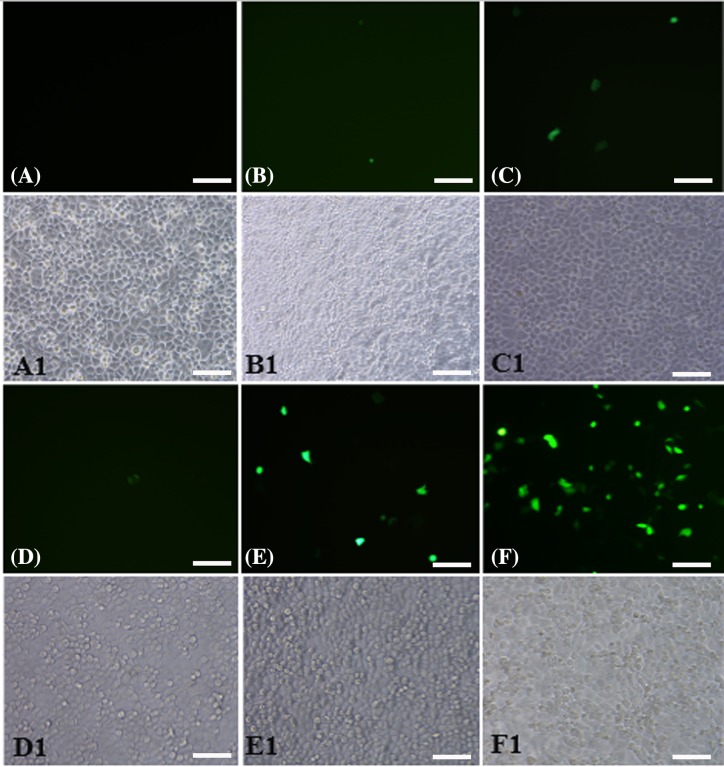
Microscopic view of transfected and adherent miR-let7b-mediated with different transfection methods to CD133^+^ OCSCs (200×) (**A**) The control group; (**B**) the plasmid group; (**C**) the SonoVue + plasmid group; (**D**) the ultrasound irradiation + plasmid group; (**E**) the ultrasound irradiation + SonoVue + plasmid group; (**F**) the liposome + plasmid group. (**A1**–**F1**) represent the observation under white light, which is corresponded to (A–F), respectively. Scale bars, 100 μm.

In this part, CD133^+^ OCSCs were transfected *in vitro* and were observed under a microscope after 48 h transfection (Figure 5): (i) the control group (C): no fluorescent expression, (ii) the plasmid group (P): visible point-like fluorescent expression and uneven distribution, (iii) SonoVue + the plasmid group (MB+P): visible point-like fluorescent expression and uneven distribution, (iv) ultrasound irradiation + plasmid group (US+P): visible little point-like fluorescent expression, more fluorescence seen in the perimeter than in the center of the plate, and less dense cells in the center than in the perimeter of the plate under white light, (v) ultrasound irradiation + SonoVue + the plasmid group (UTMD+P): significantly higher visible fluorescence expression than in the pure plasmid transfection or ultrasound, SonoVue + plasmid group, even fluorescence distribution, and (vi) liposome + plasmid group (L+P): dense and uniform fluorescence under the microscope.

### Transfection efficiency

**Figure 6:**
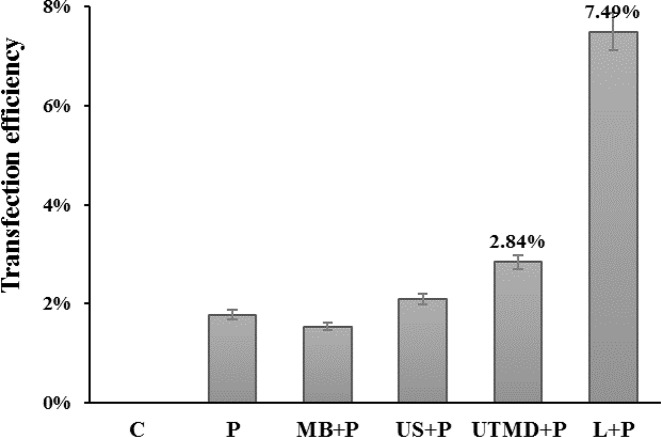
Transfection efficiency of CD133^+^ OCSCs transfected by miR-let-7b mediated with different transfection methods (**A**) The control group; (**B**) the plasmid group; (**C**) the SonoVue + plasmid group; (**D**) the ultrasound irradiation + plasmid group; (**E**) the ultrasound irradiation + SonoVue + plasmid group; (**F**) the liposome + plasmid group.

The transfection efficiency after 48 h culture in each group was tested with flow cytometry, and the results showed that the ultrasound irradiation + SonoVue group had significantly higher transfection efficiency than the pure plasmid group and the ultrasound irradiation/SonoVue + plasmid group, although less than the liposome group (Figure 6).

### Cell viability after transfection

**Figure 7:**
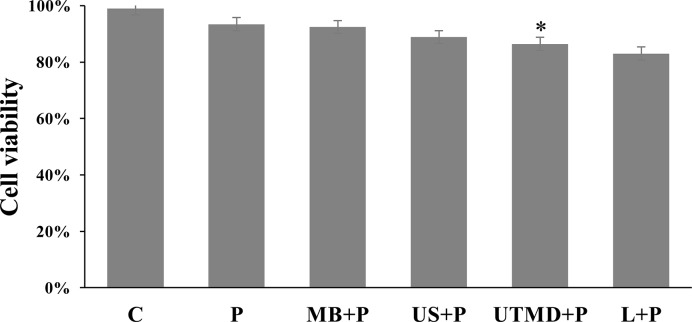
Cell viability of CD133^+^ OCSCs after different transfection methods The data are represented as means ± SD (*n*=3); *P<0.01 versus control.

The cell viability of each group was tested by the CCK8 method after miR-let-7b transfection. The results are shown in Figure 7. The UTMD group had lower cell viability than the plasmid group, ultrasound irradiation, or SonoVue plus plasmid group, and it had a poorer inhibitory effect on the proliferation of CD133^+^ OCSCs than the liposome group.

### MiR-let-7b expression in the transfected CD133^+^ OCSCs

**Figure 8:**
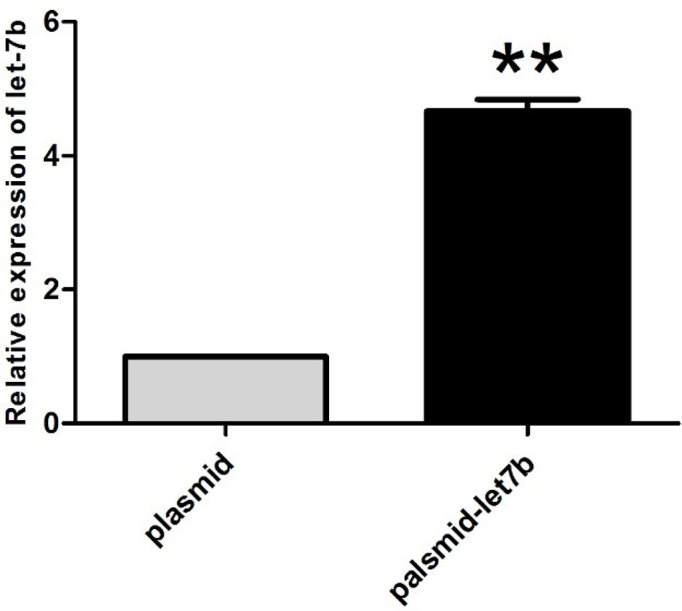
miR-let-7b expression of CD133^+^ OCSCs after transfection of miR-let-7b Relative expression of miR-let-7b in CD133^+^ OCSCs treated with miR-let-7b plasmid compared with non-treated cells. The data are represented as means ± SD (*n*=3); **P<0.001

The relative expression of miR-let-7b in CD133^+^ OCSCs of UTMD+P was analyzed compared with non-treated cells (Figure 8). It was shown that the relative expression of miR-let-7b increased almost five folds after transfection. From this result, we could confirm that the miR-let-7b plasmid was successfully delivered to CD133^+^ OCSCs.

### Apoptosis rate of CD133^+^ OCSCs after transfection

**Figure 9:**
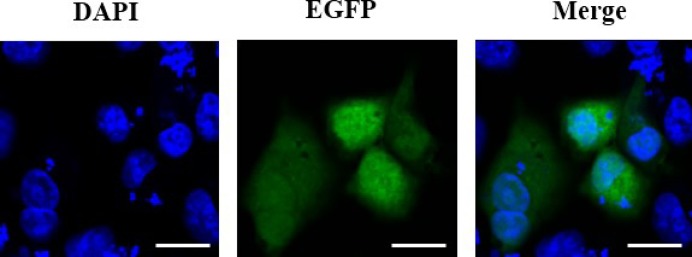
Apoptosis assessed by DAPI staining

**Figure 10:**
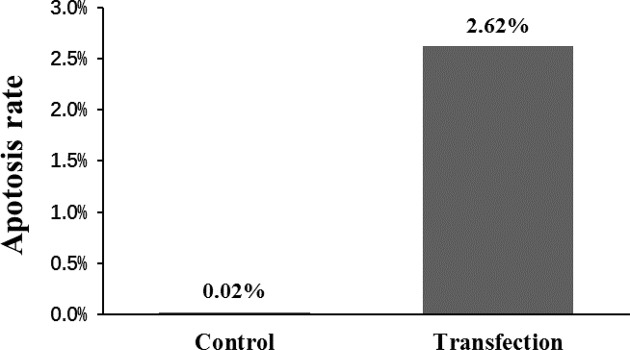
The late apoptosis and necrosis rate of CD133^+^ OCSCs after transfection of miR-let-7b using UTMD The late apoptosis and necrosis rate was estimated with flow cytometry of PI stained transfected cells.

The late apoptosis in CD133^+^ OCSCs of UTMD+P group was observed with DAPI staining under confocal laser scanning microscopy (Figure 9) and further evaluated by single-staining PI (Figure 10). As expected, transfected CD133^+^ OCSCs (2.62%) were apoptotic to some extent compared with the non-treated cells (0.02%), which did not show any obvious apoptosis. The results of flow cytometry analysis were consistent with the phenomena observed with confocal laser scanning microscopy.

### CD133^+^ expression in OCSCs

**Figure 11:**
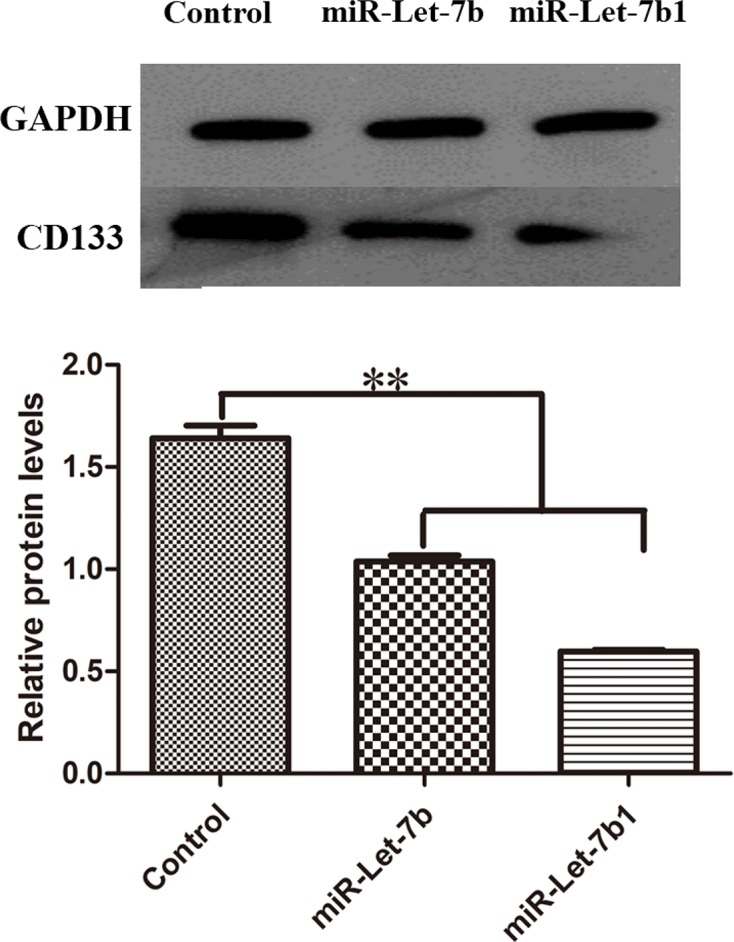
Protein expression of transfected CD133^+^ OCSCs GAPDH (glyceraldehyde 3-phosphate dehydrogenase) was used as a loading control, and relative CD133 protein levels of UTMD+P group were much lower. **P<0.01 compared with control group.

The expression of OCSC surface marker CD133 was detected by Western blot analysis (Figure 11). The results showed a decrease in CD133 expression after miR-Let-7b transfection, which indicated that miR-let-7b transfection could help to reduce the stem cell ability of CD133^+^ OCSCs.

## Discussion

Cancer statistics [1,22] indicate that ovarian cancer has the highest mortality rate amongst female reproductive system diseases and ranks as fifth amongst systemic tumors. The common therapeutic methods are aimed at normal ovarian cancer cells, but resulting in poor treatment effects due to drug resistance, tumor recurrence, or metastasis. Accumulated evidences [23] have shown that these poor outcomes are due to tumor stem cells, which are characterized by self-renewal, infinite proliferation, multidirectional differentiation in ovarian cancer cells, and are always associated with ovarian cancer occurrence, development, drug resistance, and metastasis. Thus, it is particularly important to explore a novel and effective treatment method aiming at OCSCs.

Separating enough CSCs is the premise for OCSCs study. The surface markers of OCSCs have been extensively explored. For example, CD133, ALDH1, CD44, and CD117 have been used as OCSCs markers [24]. Amongst the surface markers, CD133 has been proved to be a reliable marker for OCSCs separation in many studies [25,26]. In our study, the CD133 was used as the OCSC surface marker. And the stem cells’ characteristics of CD133^+^ OCSCs have been proved by spheroid formation assay and self-renewal ability test, which further indicated that CD133 is a reliable surface marker for OCSCs separation.

The miR-let7 family, consisting of 13 tumor suppressor genes with temporal regulation function, was discovered and confirmed for the first time in nematodes by Reinhart et al. [27]. Many researchers have explored the functions of the let-7 family and found that it plays a significant role in tumor stem cells. Thomson et al. [28] found high let-7 precursor expression levels in embryonic stem cells and low expression in differentiated cells. The present study indicated that miR-let-7 has the ability to promote differentiation of embryonic stem cells and prevent self-renewal [29]. Yu et al. [30] used lentiviral carriers of let-7 miRNAs to transfect breast CSCs, which yielded decreased proliferation and spheroid formation ability of the transfected breast CSCs, and improved differentiation ability, which indicated that promoting let-7 miRNA expression in tumor tissue can improve the therapeutic effect of tumor in many aspects. Until now, a number of studies have shown that let-7 miRNA transfection of CSC could effectively inhibit tumor proliferation, formation, and improve the level of CSC differentiation. Yang et al. [31] used liposome-mediated let-7 mimics for breast CSC transfection, and the results showed that the group with high let-7 expression contained significantly lower breast CSCs than the group with low let-7 expression. In addition, overexpression of let-7 could significantly reduce the number of spheroids formed by breast CSCs *in vitro*, whereas inhibiting let-7 expression could induce increased number of spherical cells. Liu et al. [32] conducted similar experiments in prostate cancer and came to the same conclusion; they found that let-7 inhibited tumor growth by restricting the cell proliferation in G_2_-M phase. In our study, miR-let-7b eukaryotic gene expression vector by UTMD was used to increase its expression level in OCSC, which resulting in CD133^+^ down-regulation in OCSC, decreased growth and clear apoptosis.

Recently, with the continuous development of contrast agents and ultrasound deliver technologies, the method of ultrasound targetting gene delivery has been further optimized. The cavitation effect of ultrasound combined with microbubble irradiation impacts cells, improving the cell membrane permeability or triggering recoverable holes [33] in the cell membrane, as well as promoting the absorption of genes [34]. Ultrasound targetted delivery technology has been widely used in different cancers, such as cervical cancer [35], ovarian cancer [36], breast cancer [37], and so on. Wu et al. [38] loaded NET-1 siRNA on nano-microbubbles with targetting antibodies and used low-frequency ultrasound to trigger targetting delivery, which inhibited the growth of hepatocellular carcinoma cells. The apoptotic rate of hepatocellular carcinoma cells was 14.65 ± 2.1% in this delivery method triggered by ultrasound. The transfection efficiency of NET-1 siRNA and the apoptotic rate of hepatocellular carcinoma cells were significantly increased when compared with those lacking ultrasound irradiation treatment. Wang et al. [39] prepared nanoparticles modified with PLGA-PEG and delivered miR-22-loaded nanoparticles to murine colon carcinoma under ultrasound combined SonoVue irradiation. The results showed that ultrasound irradiation could increase the expression of miR-22 by 7.9-fold.

Gene delivery based on viral vectors or chemicals has a better transfection efficiency but suffered from safety problems or immunogenic response. Compared with viral vectors or chemicals gene delivery method, UTMD is a much safer and promising approach. However, there are still a few reports about using UTMD to transfect gene into CSCs. As far as our knowledge, Liu et al. [40] used UTMD method to transfect CD133^+^ liver CSCs with shRNA and resulted in CD133 down-regulation. And some studies demonstrated that ultrasound-combined microbubbles can make the breast CSCs more sensitive to drugs such as DOX [41]. For other forms of stem cells such as mesenchymal stem cells, therapeutic ultrasound combined with Optison™ microbubbles was used to transfect with a pDNA encoding for PEX, a protein that inhibits tumor angiogenesis. And the results demonstrated ultrasound-mediated gene delivery could improve the PEX expression [42]. In our study, the intensity of fluorescence expression and flow cytometry results showed that UTMD+P group had higher transfection efficiency than the plasmid group, ultrasound irradiation group, and the microbubble and plasmid transfection group. What is more, the OCSCs result in apoptosis and show less stem cell ability after transfecting miR-let7b using UTMD. In conclusion, our study demonstrated that UTMD-mediated miR-let-7b transfection of CD133^+^ OCSCs can decrease the CD133 expression, inhibit growth, and induce apoptosis. Considering that OCSCs are an important reason for the ovarian cancer’s high recurrence and chemotherapy resistance. The UTMD-combined miRNA delivery could be a promising treatment method aiming at OCSCs, which provides an alternative option for gene therapy of ovarian cancer or combination with chemotherapy. However, it should be noted that the transfection efficiency of CSCs using UTMD is still low, further precise cavitation effects by UTMD should be explored and used to improve the gene delivery efficiency.

## Abbreviations

APC: allophycocyanin
APC-CD133: APC marked murine anti-human CD133 antibody
bFGF: basic fibroblast growth factor
CCK8: cell counting kit-8
CSC: cancer stem cell
EGF: epidermal growth factor
FACS: fluorescence activated cell sorter
OCSC: ovarian CSC
OD: optical density
PEG: polyethylene glycol
PEX: hemopexin-like domain fragment
PLGA: poly(lactic-co-glycolic acid)
qPCR: real-time quantitative PCR detecting system
UTMD: ultrasound-targetted microbubble destruction
